# Supplementary material for: Bifurcation analysis of an influenza A (H1N1) model with treatment and vaccination
Source: PLoS One. 2025 Jan 6;20(1):e0315280. doi: 10.1371/journal.pone.0315280 (PMC11703119; doi:10.1371/journal.pone.0315280)
Supplement: S1 File — (ZIP) [file pone.0315280.s002.zip › S1.pdf]

Supporting information

S1. Demonstrations of the Analytical Findings. This section represents the statements and proofs for the analytical results.

First, we review the model to understand the following theories and conceptual observations. The model for influenza transmission is defined as follows:

$$\begin{cases} \dfrac{dS}{dt} = \Lambda - (\beta_1 E + \beta_2 I)\, S - (\mu + \phi)S. \\ \dfrac{dV}{dt} = \phi S - (1-\varepsilon)\,(\beta_1 E + \beta_2 I)\, V - \mu V. \\ \dfrac{dE}{dt} = (\beta_1 E + \beta_2 I)\, S - (\alpha + \mu)E. \\ \dfrac{dI}{dt} = \alpha E + (1-\varepsilon)\,(\beta_1 E + \beta_2 I)\, V - (\mu + \delta + \gamma + \gamma_1)I. \\ \dfrac{dR}{dt} = \gamma I - \mu R. \\ \dfrac{dT}{dt} = \gamma_1 I - \mu T. \end{cases} \tag{1}$$

for $t \in (0, \infty)$ with initial conditions,

$$S(0) = S_0 \geq 0, V(0) = V_0 \geq 0, E(0) = E_0 \geq 0, I(0) = I_0 \geq 0, R(0) = R_0 \geq 0, \text{ and } T(0) = T_0 \geq [illegible] \tag{2}$$

and the total population for the SVEIRT model is found by,

$$N(t) \equiv S(t) + V(t) + E(t) + I(t) + R(t) + T(t). \tag{3}$$

Existence of Solution.

Theorem 1. *(Existence of solution) Let* $\{S_0, V_0, E_0, I_0, R_0, T_0\} \in \mathbb{R}^6$ *be presented. There subsists* $t_0 > 0$*, and continuously differentiable functions* $\{S, V, E, I, R, T : [0, t_0) \to \mathbb{R}\}$ *such that the ordered pairs of states* (S, V, E, I, R, T) *satisfies* (1) *and* $(S, V, E, I, R, T)(0) = (S_0, V_0, E_0, I_0, R_0, T_0)$.

Proof. The Picard-Lindelof Theorem asserts that concerning the initial value issue, $y'(t) = f(y(t)),\ y(t_0) = y_0,\ t \in [t_0 - \epsilon,\ t_0 + \epsilon]$, if f is continuous in t and locally Lipschitz in y, then for some value $\epsilon > 0$, a unique solution $y(t)$ exists to the initial value problem within the range $[t_0 - \epsilon, t_0 + \epsilon]$. Because the system of ODEs is autonomous, it suffices to demonstrate that the function $\mathbf{f} : \mathbb{R}^6 \to \mathbb{R}^6$ determined by,

$$\mathbf{f}(\mathbf{y}) = \begin{pmatrix} \Lambda - (\beta_1 E + \beta_2 I)S - (\mu + \phi)S \\ \phi S - (1-\varepsilon)(\beta_1 E + \beta_2 I)V - \mu V \\ (\beta_1 E + \beta_2 I)S - (\alpha + \mu)E \\ \alpha E + (1-\varepsilon)(\beta_1 E + \beta_2 I)V - (\mu + \delta + \gamma + \gamma_1)I \\ \gamma I - \mu R \\ \gamma_1 I - \mu T \end{pmatrix}$$

is locally Lipschitz in its y argument. The Jacobian matrix,

$$\nabla \mathbf{f}(\mathbf{y}) = \begin{pmatrix} a_{11} & 0 & -\beta_1 S & -\beta_2 S & 0 & 0 \\ \phi & a_{22} & -\lambda\beta_1 V & -\lambda\beta_2 V & 0 & 0 \\ \beta_1 E + \beta_2 I & 0 & a_{33} & \beta_2 S & 0 & 0 \\ 0 & a_{42} & \alpha + \lambda\beta_1 V & a_{44} & 0 & 0 \\ 0 & 0 & 0 & \gamma & -\mu & 0 \\ 0 & 0 & 0 & \gamma_1 & 0 & -\mu \end{pmatrix}$$

is linear in $\mathbf{y} \in \mathbb{R}^6$. Where

$a_{11} = -(\beta_1 E + \beta_2 I) - (\mu + \phi),\ a_{22} = -\lambda(\beta_1 E + \beta_2 I) - \mu,$

$a_{33} = \beta_1 S - (\alpha + \mu),\ a_{42} = \lambda(\beta_1 E + \beta_2 I)$, and $a_{44} = \lambda\beta_2 V - (\mu + \delta + \gamma + \gamma_1)$.

So, on a closed interval, $\nabla \mathbf{f}(\mathbf{y})$ is continuous while differentiable on an open interval $I_1 \in \mathbb{R}^6$. According to the Mean Value Theorem,

$$\frac{|\mathbf{f}(\mathbf{y_1}) - \mathbf{f}(\mathbf{y_2})|}{|\mathbf{y_1} - \mathbf{y_2}|} \le |\nabla \mathbf{f}(\mathbf{y}^*)|$$

for $\mathbf{y}^* \in I_1$. Let $|\nabla \mathbf{f}(\mathbf{y}^*)| = K$, we get $|\mathbf{f}(\mathbf{y_1}) - \mathbf{f}(\mathbf{y_2})| \le K|\mathbf{y_1} - \mathbf{y_2}|$ for all $\mathbf{y_1}, \mathbf{y_2} \in I_1$, and therefore for every $\mathbf{y} \in \mathbb{R}^6$, $\mathbf{f}(\mathbf{y})$ is locally bounded.As a result, $\mathbf{f}$ is locally Lipschitz in $\mathbf{y}$ since it has a continuous, bounded derivative on any compact subset $\mathbb{R}^3$. The Pichard-Lindelof theorem states that for any time $t_0 > 0$, there is a unique solution, $y(t)$, to the ordinary differential equation $y'(t) = f(y(t))$ with starting value $y(0) = y_0$ on $[0, t_0]$. □

Positivity of Solution. The boundedness and positivity of the solutions are two main constituents of an epidemic model. To convey that any solution with positive beginning values stays positive for all times $t > 0$, it is necessary to establish that all parameters and variables are always positive for $t > 0$. Positive behavior is biologically interpreted as the long-term survival of the population [1, 2].

Theorem 2. *(Positivity of solution) Consider the initial conditions of the system* (1) *are* $S(0) \ge 0,\ V(0) \ge 0,\ E(0) \ge 0,\ I(0) \ge 0,\ R(0) \ge 0,$ *and* $T(0) \ge 0$*; the solutions* $S(t),\ V(t),\ E(t),\ I(t),\ R(t),$ *and* $T(t)$ *are non negative* $\forall\, t > 0$.

Proof. Assume that

$$\widehat{t} = \sup\{t > 0 : S(t) \ge 0, V(t) \ge 0, E(t) \ge 0, I(t) \ge 0, R(t) \ge 0, \text{and } T(t) \ge 0\} \in [0, t].$$

Since the solution is continuous and each initial condition is non-negative, there must be a period while the outcome is still positive, and we observe that $\widetilde{t} > 0$. Then, each term is calculated on the interval $[0, t]$. Thus, $\widehat{t} > 0$ and results from the equation of system (1) that,

$$\frac{\mathrm{d}S}{\mathrm{d}t} \ge \Lambda - (\lambda_1 + \mu)S. \quad [\text{where } \lambda_1 = (\beta_1 E + \beta_2 I)]$$

This inequality can be resolved by applying the integrating factor approach.

$$\frac{d}{dt}\left\{S(t)\exp\left[\mu(t) + \int_0^t \lambda_1(s)dS\right]\right\} \ge \pi \exp\left[\mu t + \int_0^t \lambda_1(s)dS\right].$$

Integrating both sides yields,

$$S(\widehat{t})\exp\left[\mu\widehat{t} + \int_0^{\widehat{t}} \lambda_1(s)dS\right] \ge \int_0^{\widehat{t}} \pi \exp\left[\mu\widehat{t} + \int_0^{\widehat{t}} (\lambda_1(w))dw\right] d\widehat{t} + C.$$

Where C is the integration constant depending on the upper limit of $\lambda_1,\ \mu$, and $S(0)$. Hence,

$$S(\widehat{t}) \geq S(0)\exp\left[-\left(\mu\widehat{(t)} + \int_0^{\widehat{t}}(\lambda_1(S)dS)\right)\right] +$$
$$\exp\left[-\left(\mu\widehat{(t)} + \int_0^{\widehat{t}}(\lambda_1(S)dS)\right)\right] \boldsymbol{.} \left(\int_0^{\widehat{t}}\pi\exp\left[(\mu\widehat{(t)} + \int_0^{\widehat{t}}(\lambda_1(w)dw))\right]d\widehat{t}\right) > 0.$$

So, $S(\widehat{t}) \geq 0,\ \forall\,\widehat{t} \geq 0$.

Next, from the positivity of the solutions place bounds on other compartments [1]. Here,

$$\frac{dV}{dt} \geq -\left((1-\varepsilon)\lambda_1 + \mu\right)V$$
$$\Rightarrow V(\widehat{t}) \geq V(0)\exp\left[-\left\{\mu\widehat{t} + \int_0^{\widehat{t}}(1-\varepsilon)\lambda_1(s)dS\right\}\right] > 0,\ \forall\,\widehat{t} \geq 0.$$

It is examined that,

$$E(\widehat{t}) \geq E(0)e^{-(\mu+\alpha)\widehat{t}} > 0.$$
$$I(\widehat{t}) \geq I(0)e^{-(\mu+\delta+\gamma+\gamma_1)\widehat{t}} > 0.$$
$$R(\widehat{t}) \geq R(0)e^{-\mu\widehat{t}} > 0.$$
$$T(\widehat{t}) \geq T(0)e^{-\mu\widehat{t}} > 0.$$

for $\widetilde{t} \in [0,t]$. As a result, an upper limit can be set for $S(t), V(t), E(t), I(t), R(t)$, and $T(t)$. Therefore, all the solutions of the system (1) will stay non-negative for $t \geq 0$, encompassing at time $\widetilde{t}$. According to continuity, there must exist $t > \widetilde{t}$ such that $S(t), V(t), E(t), I(t), R(t)$, and $T(t)$ are strictly positive on the entire interval $[0,t]$. Further extending the interval of existence is possible because all functions stay bounded on this same interval [2]. The bounds on the compartments S, V, E, I, R, and T that were derived previously hold for any brief time (compact interval). Consequently, we can extend the existence of the solution to $[0,t]$ for every $t > 0$. Based on the aforementioned reasoning, the solutions continue to be positive and confined to $[0,t]$. □

Boundedness of Solution. Boundedness can be understood as a natural growth constraint resulting from scarce resources, while positivity suggests that every member of the compartment population survives [1, 4–6].

Theorem 3. *[2]. (Positive invariance and boundedness of solutions) The closed region* $\Omega = \{(S,V,E,I,R,T) \in \mathbb{R}^6_+ : 0 < N \leq \frac{\Lambda}{\mu}\}$ *is positively invariant and attracting set for the system (1).*

Proof. We can write in vector form,

$$X = (S,V,E,I,R,T)^T \in \mathbb{R}^6.$$

We define,

$$F(X) = \begin{pmatrix} F_1(X) \\ F_2(X) \\ F_3(X) \\ F_4(X) \\ F_5(X) \\ F_6(X) \end{pmatrix} = \begin{pmatrix} \Lambda - (\beta_1 E(t) + \beta_2 I(t))S(t) - (\mu + \phi)S(t) \\ \phi S(t) - (1-\varepsilon)(\beta_1 E(t) + \beta_2 I(t))V(t) - \mu V(t) \\ (\beta_1 E(t) + \beta_2 I(t))S(t) - (\alpha + \mu)E(t) \\ \alpha E(t) + (1-\varepsilon)(\beta_1 E(t) + \beta_2 I(t))V(t) - (\mu + \delta + \gamma + \gamma_1)I(t) \\ \gamma I(t) - \mu R(t) \\ \gamma_1 I(t) - \mu T(t) \end{pmatrix}$$

where $F : \mathbf{C}_+ \to \mathbb{R}^6$, and $F \in \mathbf{C}^\infty(\mathbb{R}^6)$.
Now,

$$\dot{X} = F(X_t),$$

where $\boldsymbol{.} \equiv \dfrac{d}{dt}$ including $X_t(\theta) = X(t+\theta),\ \theta \in [0, \tau]$.

It is simple to verify that whenever we desire $X(\theta) \in \mathbf{C}_+$ such that $X_i = 0$, then we acquire $F_i(X)|_{X_i(t)=0},\ X_t \in \mathbf{C}_+ \geq 0,\ i = 1, 2, \cdots, 6$. Any result to the model's equation, alongside $X_t(\theta) \in \mathbf{C}_+$ say, $X(t) = X(t, X(0))$ is such that $X(t) \in \mathbb{R}^6_{0+}$ for all $t > 0$. The size of the population, $N = S + V + E + I + R + T$ with the initial conditions, $S(0) \geq 0,\ V(0) \geq 0,\ E(0) \geq 0,\ I(0) \geq 0,\ R(0) \geq 0,\ T(0) \geq 0.$

Now, for the boundedness of the solution we define,

$$\frac{dN}{dt} = \leq \Lambda - \mu N.$$

This indicates that $N(t)$ is bounded, and so are the $S(t), V(t), E(t), I(t), R(t)$ and $T(t)$.

$$\text{Here,} \quad N \leq N_0 e^{-\mu t} + \frac{\Lambda}{\mu}\left(1 - e^{-\mu t}\right)$$

from this expression when $t \to \infty$, and $N(t) \leq \dfrac{\Lambda}{\mu}$. The system will be examined in biologically feasible regions as follows. As a result, we can consider the feasible region $\Omega = \left\{(S, V, E, I, R, T) \in \mathbb{R}^6_+ : S \leq \dfrac{\Lambda}{\mu}, V, E, I, R, T \geq 0\right\}$.

□

Theorem 4. *[2]. The feasible region* Ω *is determined by,*

$$\Omega = \left\{(S(t),\ V(t),\ E(t),\ I(t),\ R(t),\ T(t)) \in \mathbb{R}^6_+ \ \mid \ 0 \leq N \leq \max\left\{N(0), \frac{\Lambda}{\mu}\right\}\right\}.$$

with initial conditions $S(t) > 0,\ V(t) > 0,\ E(t) > 0,\ I(t) > 0,\ R(t) > 0,\ T(t) > 0,$ *is positively invariant and attracting with respect to system (1)* $\forall\ t > 0$.

Proof. Here,

$$\frac{dN}{dt} = \Lambda - \mu N.$$

The omission of the influenza infection ensures that,

$$\frac{dN}{dt} \leq \Lambda - \mu N.$$

Now,

$$N(t) \leq \frac{\Lambda}{\mu} + \left(N(0) - \frac{\Lambda}{\mu}\right)\exp(-\mu t). \tag{4}$$

From (4), we examine that as $t \to \infty,\ N(t) \to \dfrac{\Lambda}{\mu}$. So, if $N(0) \leq \dfrac{\Lambda}{\mu}$ then $\lim_{t\to\infty} N(t) = \dfrac{\Lambda}{\mu}$. On the other hand, if $N(0) > \dfrac{\Lambda}{\mu}$, then total population N will decrease to $\dfrac{\Lambda}{\mu}$ as $t \to \infty$. Particularly, $N(t) < \dfrac{\Lambda}{\mu}$ if $N(0) < \dfrac{\Lambda}{\mu}$. This means that $N(t) \leq \max\left\{N(0), \dfrac{\Lambda}{\mu}\right\}$. Hence, no solution path crosses any borders of Ω, making the region Ω a positively invariant set of the model (1). This demonstrates that the developed model is applicable from a mathematical and epidemiological perspective [1,3]. The model is appraised in the biologically feasible region which means the considered model is well-defined. Further, $N(t) > \dfrac{\Lambda}{\mu}$, then the other solution enters Ω in finite time, or $N(t)$ approaches to $\dfrac{\Lambda}{\mu}$, and the variables $E(t),\ I(t),\ R(t),\ V(t)$ and $T(t)$ approach to zero. Hence the region Ω is attracting.

Therefore, $N(t)$ is bounded above. Subsequently, $S(t),\ V(t),\ E(t),\ I(t),\ R(t)$ and $T(t)$ are all bounded above. Thus, in Ω, system (1) is a well-posed and global attractor of the system. □

Proof of Theorem 4. Utilizing Lemma 1 on the model (1), we acknowledge that $X_1 = (S, R)$ and $X_2 = (E, I)$ when the system at the DFE. At this DFE, the state variables are given by $X_1^* = (N, 0)$. It is significant to remember that,

$$\frac{dX_1}{dt} = F(X_1, 0) = \begin{pmatrix} \mu N - (\mu + \phi)S \\ -\mu R \end{pmatrix}$$

is linear and its result can be easily identified as,

$$R(t) = R(0)e^{-\mu t},\ S(t) = N - (N - S(0))e^{-\mu t}.$$

Evidently, as $t \to \infty$, both $R(t)$ tends to 0 and $S(t)$ tends to N, regardless of the initial values of $R(0)$ and $S(0)$. Thus, the equilibrium point $X_1^* = (N, 0)$ is globally asymptotically stable, and condition (H1) is satisfied. Next,

$$G(X_1, X_2) = \begin{pmatrix} (\beta_1 E + \beta_2 I)S - (\alpha + \mu)E \\ \alpha E - (\mu + \delta + \gamma + \gamma_1)I \end{pmatrix}.$$

We can obtain,

$$A = \begin{pmatrix} \beta_1 N - (\alpha + \mu) & \beta_2 N \\ \alpha & -(\mu + \delta + \gamma + \gamma_1) \end{pmatrix}$$

with all non-negative off-diagonal elements. Consequently,

$$\hat{G}(X_1, X_2) = \begin{pmatrix} \beta_1 E(N - S) + \beta_2 I(N - S) \\ 0 \end{pmatrix}.$$

Since, $0 \leq S \leq N$, it is obvious that $\hat{G} \geq 0$. That leads to the global stability of DFE for $\mathcal{R}_0 < 1$.

Proof of Theorem 5. To examine the global stability of $\mathcal{E}^0$, we assume a Lyapunov functional $U_1(t)$,

$$U_1 = \bar{S}F\left(\frac{S}{\bar{S}}\right) + E + I = \left(S - \bar{S} - \bar{S}\ln\frac{S}{\bar{S}}\right) + E + I.$$

Here, U_1 is continuous, well-defined, and positive definite for all $(S, V, E, I, R, T) > 0$ and $\theta \in [0, \tau]$.

It illustrates that U_1 is always non-negative, and U_1 equals zero exclusively when assessed at the non-infective equilibrium point $\mathcal{E}^0$. Moreover, the global minimum of U_1 is achieved at $\mathcal{E}^0$. Consequently, all outcomes converge toward the infection-free steady state $\mathcal{E}^0$. Additionally, the functions U_1 along the system's trajectories adhere to the following relations:

$$\frac{dU_1}{dt} = \left(1 - \frac{\bar{S}}{S}\right)(\Lambda - (\beta_1 E + \beta_2 I)S - (\mu + \phi)S) + (\beta_1 E + \beta_2 I)S - (\mu + \alpha)E + \alpha E + \lambda(\beta_1 E + \beta_2 I)V - (\mu + \delta + \gamma + \gamma_1)I.$$

Utilizing the infection-free steady state of the model (1), $\Lambda = (\mu + \phi)\bar{S}$ in above expression, then the equation becomes,

$$\begin{aligned}\frac{dU_1}{dt} &\leq -\frac{(\mu+\phi)}{S}(S-\bar{S})^2 + \beta_1 E\bar{S} + \beta_2 I\bar{S} - (\mu+\phi)E + \alpha E - (\mu+\delta+\gamma+\gamma_1)I \\ &\leq -\frac{(\mu+\phi)}{S}(S-\bar{S})^2 + \left(\frac{\alpha}{\alpha+\mu}\left(\frac{S_0\left[\alpha\beta_2 + \beta_1(\gamma+\gamma_1+\mu+\delta)\right]}{(\alpha+\mu)(\gamma+\gamma_1+\delta+\mu)}\right) - 1\right)(\mu+\delta+\gamma+\gamma_1)I - (\beta_1\bar{S} - \mu)E \\ &\leq -\frac{(\mu+\phi)}{S}(S-\bar{S})^2 + \left(\frac{\alpha}{\alpha+\mu}\mathcal{R}_0 - 1\right)(\mu+\delta+\gamma+\gamma_1)I - \left(\beta_1\frac{\mu N}{\mu+\phi} - \mu\right)E \\ &\leq 0.\end{aligned}$$

Here, we substituted the DFE value $S_0 = \frac{\mu N}{\mu + \phi}$. If $\mathcal{R}_0 < 1$, then $\frac{dU_1}{dt}$ is negative. Additionally, $\frac{dU_1}{dt} = 0$ if and only if $S(t) = \bar{S}$ and $E(t) = I(t) = R(t) = 0$. Therefore, based on the Lasalle invariance principle, the infection-free equilibrium point $\mathcal{E}^0$ is globally asymptotically stable over Ω in the scenario where $\mathcal{R}_0 < 1$.

References

1. Kanyiri CW, Mark K, Luboobi L. Mathematical analysis of influenza A dynamics in the emergence of drug resistance. Computational and Mathematical Methods in Medicine. 2018;2018(1):2434560.

2. Krishnapriya P, Pitchaimani M, Witten TM. Mathematical analysis of an influenza A epidemic model with discrete delay. Journal of computational and Applied Mathematics. 2017 Nov 1;324:155-72.

3. Khanh NH. Stability analysis of an influenza virus model with disease resistance. Journal of the Egyptian Mathematical Society. 2016 Apr 1;24(2):193-9.

4. Akhi AA, Tasnim F, Akter S, Kamrujjaman M. A MATHEMATICAL MODEL OF A DIPHTHERIA OUTBREAK IN ROHINGYA SETTLEMENT IN BANGLADESH. Journal of Mahani Mathematical Research Center. 2023 Jul 1;12(2).

5. Akhi AA, Kamrujjaman M, Nipa KF, Khan T. A continuous-time Markov chain and stochastic differential equations approach for modeling malaria propagation. Healthcare Analytics. 2023 Dec 1;4:100239.

6. Akhi AA, Mohammad KM, Kamrujjaman M. Seasonal variability and stochastic branching process in malaria outbreak probability. Acta Tropica. 2024 Sep 1;257:107311.
