# Supplementary material for: Bifurcation analysis of an influenza A (H1N1) model with treatment and vaccination
Source: PLoS One. 2025 Jan 6;20(1):e0315280. doi: 10.1371/journal.pone.0315280 (PMC11703119; doi:10.1371/journal.pone.0315280)
Supplement: S2 File — (ZIP) [file pone.0315280.s003.zip › S2.pdf]

Supporting information

S2. The Endemic Equilibrium Point. This appendix presents the brief calculation for computing fixed points untitled the section "Determination of Fixed Points".

The variables for the EE are substituted as $(\widetilde{S},\widetilde{V},\widetilde{E},\widetilde{I},\widetilde{R},\widetilde{T}) \equiv (S^*,V^*,E^*,I^*,R^*,T^*)$, where $E^*>0$, and $I^*>0$ also $E^*\neq 0,\ I^*\neq 0$. And we have the following system as follows,

$$\begin{cases} \Lambda - (\beta_1 E^* + \beta_2 I^*)\, S^* - (\mu+\phi) S^* = 0. \\ \phi S^* - (1-\varepsilon)\,(\beta_1 E^* + \beta_2 I^*)\, V^* - \mu V^* = 0. \\ (\beta_1 E^* + \beta_2 I^*)\, S^* - (\alpha+\mu) E^* = 0. \\ \alpha E^* + (1-\varepsilon)\,(\beta_1 E^* + \beta_2 I^*)\, V^* - (\mu+\delta+\gamma+\gamma_1) I^* = 0. \\ \gamma I^* - \mu R^* = 0. \\ \gamma_1 I^* - \mu T^* = 0. \end{cases} \tag{1}$$

Now, adding the first and third equation of (1) we have,

$$\begin{cases} \Lambda - (\mu+\phi)S^* = (\alpha+\mu)E^* \\ \Rightarrow S^* = \dfrac{\Lambda - (\alpha+\mu)E^*}{(\mu+\phi)} = \dfrac{\Lambda - a_1 E^*}{a_2}. \end{cases} \tag{2}$$

Here, $a_1 = \alpha+\mu$, and $a_2 = \mu+\phi$. Now, from the second equation of (1) we have,

$$\begin{cases} \phi S^* = \{\mu + \lambda(\beta_1 E + \beta_2 I)\}\, V^* \quad \text{where } \lambda = (1-\varepsilon) \\ \Rightarrow V^* = \dfrac{\phi S^*}{\mu + \lambda(\beta_1 E^* + \beta_2 I^*)} = \dfrac{\phi\left(\frac{\Lambda - a_1 E^*}{a_2}\right)}{\mu + \lambda(\beta_1 E^* + \beta_2 I^*)} \\ \Rightarrow V^* = \dfrac{\phi(\Lambda - a_1 E^*)}{a_2\,\{\mu + \lambda(\beta_1 E^* + \beta_2 I^*)\}}. \end{cases} \tag{3}$$

Now, from (2) substituting the value of S^*, we get from the third equation of (1),

$$\begin{aligned} &\Rightarrow S^*(\beta_1 E^* + \beta_2 I^*) = (\alpha+\mu)E^* = a_1 E^* \\ &\Rightarrow \left(\frac{\Lambda - a_1 E^*}{a_2}\right)(\beta_1 E^* + \beta_2 I^*) = a_1 E^* \\ &\Rightarrow a_1\beta_1 E^{*2} + E(a_1 a_2 + a_1\beta_2 I^* - \Lambda\beta_1) - \Lambda\beta_2 I^* = 0. \end{aligned}$$

Now,

$$E^* = \frac{(\Lambda\beta_1 - a_1 a_2 - a_1\beta_2 I^*) \pm \sqrt{(\Lambda\beta_1 - a_1 a_2 - a_1\beta_2 I^*)^2 + 4\Lambda\beta_2 I^* a_1\beta_1}}{2a_1\beta_1}. \tag{4}$$

Two roots of the EE point of (4) will be real if and only if,

$$(\Lambda\beta_1 - a_1 a_2 - a_1\beta_2 I^*)^2 > -4\Lambda\beta_2 I^* a_1\beta_1.$$

Here, from the expression of (4), one root will be always positive, other will be positive if and only if $4\Lambda\beta_2 I^* a_1\beta_1 < 0$. Now,

$$\frac{S_0\alpha\beta_2 + S_0\beta_1(\gamma+\gamma_1+\delta+\mu) + V_0\beta_2\lambda(\alpha+\mu)}{(\alpha+\mu)(\gamma+\gamma_1+\delta+\mu)} > 1$$
$$\Rightarrow S_0\alpha\beta_2 + S_0\beta_1 a_3 + V_0\beta_2\lambda a_1 > a_1 a_3.$$

Where we let $a_3 = (\gamma+\gamma_1+\delta+\mu)$. Now, from the second equation of (1) we have,

$$V^* = \frac{\phi(\Lambda - a_1E^*)}{a_2(\mu+\lambda\lambda_1)}.$$

Where we let $\lambda_1 = \beta_1E^* + \beta_2I^*$, which is the force of infection of the model (1). Similarly,

$$R^* = \frac{\lambda I^*}{\mu}, \text{ and } T^* = \frac{\lambda_1 I^*}{\mu}.$$

Thus, at the endemic equilibrium $S^*, V^*, E^*, I^*, R^*,$ and T^* depends on the nature of I^*. Now, putting all expressions from above from the third equation of (1) we have,

$$(\beta_1E^*+\beta_2I^*)S^* = (\alpha+\mu)E^* \Rightarrow \frac{(\alpha+\mu)E^* - \beta_1E^*S^*}{\beta_2S^*} = I \Rightarrow \frac{(\alpha+\mu)E^* - \beta_1E^*\left(\frac{\Lambda-a_1E^*}{a_2}\right)}{\beta_2\left(\frac{\Lambda-a_1E^*}{a_2}\right)} =$$
$$\Rightarrow a_1E^* - \beta_1E^*\left(\frac{\Lambda-a_1E^*}{a_2}\right) - \beta_2I^*\left(\frac{\Lambda-a_1E^*}{a_2}\right) = \tag{5}$$

Now, from the fourth equation of (1) we have,

$$\begin{cases} \alpha E^* + \lambda(\beta_1E^*+\beta_2I^*)V^* - (\mu+\delta+\gamma+\gamma_1)I^* = 0. \\ \alpha E^* + \lambda(\beta_1E^*+\beta_2I^*)\left\{\frac{\phi(\lambda-a_1E^*)}{a_2\{\mu+\lambda(\beta_1E^*+\beta_2I^*)\}}\right\} - (\mu+\delta+\gamma+\gamma_1)I^* = 0. \end{cases} \tag{6}$$

Let, the force of infection

$$\lambda_1 = (\beta_1E^*+\beta_2I^*),\ a_3 = (\mu+\delta+\gamma+\gamma_1), \text{ and } a_4 = (\mu+\lambda\lambda_1).$$

Now, by solving and simplifying the above two expressions using Mathematica we have obtained,

$$I^* = \frac{G_1+G_2}{(2a_1a_2a_3a_4(-a_2a_4(a_3\beta_1+\alpha\beta_2)+a_1\beta_2\lambda\lambda_1\phi))}.$$

Where
$\mathbb{K} = (a_2^2a_4(-2a_1a_2a_3a_4(a_3\beta_1+\alpha\beta_2)\Lambda + a_4(a_3\beta_1+\alpha\beta_2)^2\Lambda^2 + a_1^2a_3(a_2^2a_3a_4 + 4\beta_2\lambda\Lambda\lambda_1\phi))),$
$G_1 = -(a_1^2a_2^2a_3a_4\lambda\lambda_1 + a_2a_4\alpha(a_2a_4(a_3\beta_1+\alpha\beta_2)\Lambda + \sqrt{\mathbb{K}})),$ and
$G_2 = a_1(a_2^3a_3a_4^2\alpha + a_2a_4(-a_3\beta_1+\alpha\beta_2)\lambda\Lambda\lambda_1\phi + \lambda\lambda_1\phi\sqrt{\mathbb{K}}).$

Thus, for the threshold parameter $\mathcal{R}_0 > 1$ we have,

$$a_4(a_3\beta_1+\alpha\beta_2)^2\Lambda^2 + a_1^2a_3(a_2^2a_3a_4 + 4\beta_2\lambda\Lambda\lambda_1\phi) > 2a_1a_2a_3a_4(a_3\beta_1+\alpha\beta_2)\Lambda.$$

Hence, the expression of the EE point of the model (1) is obtained.

Since different virus particles and infected cells are present in varied amounts, we refer to this as vital persistence. We can also shorten the point as $\mathcal{E}^* = (S^*, V^*, E^*, I^*, R^*, T^*)$.

In mathematical biology, $\mathcal{E}^0$ represents a short-lived infection that naturally clears from the body. In contrast, $\mathcal{E}^*$ signifies a situation where the body can't eliminate the illness on its own. In this case, the influenza infection becomes more noteworthy over time [1, 2]. Consequently, more sophisticated models accounting for latent infection, the impact of macrophages, the cytotoxic immune response (CLT), or spatial dependence become essential to explain the dynamics of influenza spread throughout the body and its evolution toward an outbreak.

If the system explained by (1) reaches an equilibrium point, it will persist throughout the remaining period. Alternatively, the system is not required to reach these equilibrium values. However, it may approach the equilibrium, deviate from it, or oscillate between definite values. Conducting a comprehensive stability study of the system is essential for precisely predicting its behavior and understanding how it will interact with the equilibrium.

References

1. Khanh NH. Stability analysis of an influenza virus model with disease resistance. Journal of the Egyptian Mathematical Society. 2016 Apr 1;24(2):193-9.

2. Ojo M, Akinpelu F. Lyapunov functions and global properties of seir epidemic model. Int. J. Chem. Math. Phys. 2017 Mar;1(1):11-6.
