# Supplementary material for: Bifurcation analysis of an influenza A (H1N1) model with treatment and vaccination
Source: PLoS One. 2025 Jan 6;20(1):e0315280. doi: 10.1371/journal.pone.0315280 (PMC11703119; doi:10.1371/journal.pone.0315280)
Supplement: S3 File — (ZIP) [file pone.0315280.s004.zip › S3.pdf]

Supporting information

S3. Basic Reproduction Number (BRN). A brief illustration of BRN for both controlling and without controlling strategies is included here for section titled "Basic Reproduction Number and the Presence of Endemic Equilibrium".

Concise computation of BRN with control. From the model (1), in the presence of vaccination class $\mathcal{R}_{0V}$ is known as basic reproduction number with control. With vaccination the DFE of (4) is,

$$\mathcal{E}^0 \equiv \left(\frac{\Lambda}{\mu+\phi}, \frac{\phi\Lambda}{\mu(\mu+\phi)}, 0, 0, 0, 0\right).$$

We now apply the next generation matrix method to the model (1) and modeling only the exposed and infected compartments $E(t)$ and $I(t)$ is necessary since we are only interested in cells that disseminate infection. Hence, considering subpopulation $E(t)$ and $I(t)$ containing new infection terms and disease transmission terms, we can obtain the following subsystem,

$$\begin{aligned}\frac{dE}{dt} &= (\beta_1 E + \beta_2 I)S - (\alpha+\mu)E.\\ \frac{dI}{dt} &= \alpha E + (1-\varepsilon)(\beta_1 E + \beta_2 I)V - (\mu+\delta+\gamma+\gamma_1)I.\end{aligned} \tag{1}$$

From the system (1), we obtain,

$$F = \begin{pmatrix} \beta_1 S_0 & \beta_2 S_0 \\ \lambda\beta_1 V_0 & \lambda\beta_2 V_0 \end{pmatrix}, \text{ and } V = \begin{pmatrix} \mu+\alpha & 0 \\ -\alpha & \mu+\delta+\gamma+\gamma_1 \end{pmatrix}.$$

Therefore,

$$V^{-1} = \begin{pmatrix} \dfrac{1}{\alpha+\mu} & 0 \\ \dfrac{\alpha}{(\alpha+\mu)(\mu+\delta+\gamma+\gamma_1)} & \dfrac{1}{(\mu+\delta+\gamma+\gamma_1)} \end{pmatrix}.$$

Here, F and V stand for the new infection term and transferred terms, respectively. Thus, the next-generation matrix FV^{-1} is,

$$FV^{-1} = \begin{pmatrix} \dfrac{S_0\beta_1}{(\alpha+\mu)} + \dfrac{S_0\alpha\beta_2}{(\alpha+\mu)(\mu+\delta+\gamma+\gamma_1)} & \dfrac{S_0\beta_2}{(\mu+\delta+\gamma+\gamma_1)} \\ \dfrac{V_0\beta_1\lambda}{\alpha+\mu} + \dfrac{V_0\alpha\beta_2\lambda}{(\alpha+\mu)(\mu+\delta+\gamma+\gamma_1)} & \dfrac{V_0\beta_2\lambda}{(\mu+\delta+\gamma+\gamma_1)} \end{pmatrix}.$$

Here, the eigenvalues of FV^{-1} are
$\left\{0, \dfrac{S_0\alpha\beta_2 + S_0\beta_1\gamma + S_0\beta_1\gamma_1 + S_0\beta_1\delta + V_0\alpha\beta_2\lambda + S_0\beta_1\mu + V_0\beta_2\lambda\mu}{(\alpha+\mu)(\mu+\delta+\gamma+\gamma_1)}\right\}.$

Hence, the controlled basic reproduction number $\mathcal{R}_{0V}$ which is the spectral radius of FV^{-1} is given as follows,

$$\begin{aligned}\mathcal{R}_{0V} = \rho(FV^{-1}) &= \frac{S_0\alpha\beta_2 + S_0\beta_1\gamma + S_0\beta_1\gamma_1 + S_0\beta_1\delta + V_0\alpha\beta_2\lambda + S_0\beta_1\mu + V_0\beta_2\lambda\mu}{(\alpha+\mu)(\mu+\delta+\gamma+\gamma_1)}\\ &= \frac{\Lambda\left[\alpha\beta_2 + \beta_1(\mu+\delta+\gamma+\gamma_1)\right]}{(\mu+\phi)(\alpha+\mu)(\mu+\delta+\gamma+\gamma_1)} + \frac{\Lambda\phi\beta_2\lambda}{\mu(\mu+\phi)(\mu+\delta+\gamma+\gamma_1)}.\end{aligned} \tag{2}$$

Concise computation of BRN without control. From the model (1), in absence of vaccination class i.e. when $\lambda = (1-\varepsilon) = 0$, $\varepsilon = 1$, then the threshold quantity $\mathcal{R}_{0V}$ becomes $\mathcal{R}_0$ which is known as basic reproduction number without control. Without vaccination the DFE of (4) is, $\mathcal{E}^0 \equiv \left(\frac{\Lambda}{\mu+\phi}, \frac{\phi\Lambda}{\mu(\mu+\phi)}, 0, 0, 0, 0\right)$. We can extract the two following matrices from the system (1) which are F and V by replacing $\varepsilon = 0$. They are presented as follows,

$$F = \begin{pmatrix} \beta_1 S_0 & \beta_2 S_0 \\ 0 & 0 \end{pmatrix}, \text{ and } V = \begin{pmatrix} \mu+\alpha & 0 \\ -\alpha & \mu+\delta+\gamma+\gamma_1 \end{pmatrix}.$$

Thus,

$$V^{-1} = \begin{pmatrix} \frac{1}{\alpha+\mu} & 0 \\ \frac{\alpha}{(\alpha+\mu)(\mu+\delta+\gamma+\gamma_1)} & \frac{1}{(\mu+\delta+\gamma+\gamma_1)} \end{pmatrix}.$$

Here, F and V stand for the new infection term and transferred terms, respectively. Thus, the next-generation matrix FV^{-1} is,

$$FV^{-1} = \begin{pmatrix} \frac{S_0\beta_1}{(\alpha+\mu)} + \frac{S_0\alpha\beta_2}{(\alpha+\mu)(\mu+\delta+\gamma+\gamma_1)} & \frac{S_0\beta_2}{(\mu+\delta+\gamma+\gamma_1)} \\ 0 & 0 \end{pmatrix}.$$

Here, the eigenvalues of FV^{-1} are $\left\{0, \frac{S_0\alpha\beta_2 + S_0\beta_1\gamma + S_0\beta_1\gamma_1 + S_0\beta_1\delta + S_0\beta_1\mu}{(\alpha+\mu)(\mu+\delta+\gamma+\gamma_1)}\right\}$.

Hence, the basic reproduction number $\mathcal{R}_0$ which is the spectral radious of FV^{-1} is given by,

$$\begin{aligned} \mathcal{R}_0 = \rho(FV^{-1}) &= \frac{S_0\alpha\beta_2 + S_0\beta_1\gamma + S_0\beta_1\gamma_1 + S_0\beta_1\delta + S_0\beta_1\mu}{(\alpha+\mu)(\mu+\delta+\gamma+\gamma_1)} \\ &= \frac{\Lambda\left[\alpha\beta_2 + \beta_1(\gamma+\gamma_1+\mu+\delta)\right]}{(\mu+\phi)(\alpha+\mu)(\gamma+\gamma_1+\delta+\mu)}. \end{aligned} \tag{3}$$

The basic reproduction number ($\mathcal{R}_0$) is defined as the average number of secondary infections resulting from the introduction of a single virus cell into a host where every target cell is susceptible. In our model, $\mathcal{R}_0$ depends on two variables: the average number of target cells per unit of time (considering natural death) and the rate of disease transmission by an infective cell [1, 2].

References

1. Kumar Ghosh J, Saha P, Kamrujjaman M, Ghosh U. Transmission dynamics of COVID-19 with saturated treatment: a case study of Spain. Brazilian Journal of Physics. 2023 Jun;53(3):54.

2. Song H, Wang R, Liu S, Jin Z, He D. Global stability and optimal control for a COVID-19 model with vaccination and isolation delays. Results in physics. 2022 Nov 1;42:106011.
