# Supplementary material for: Bifurcation analysis of an influenza A (H1N1) model with treatment and vaccination
Source: PLoS One. 2025 Jan 6;20(1):e0315280. doi: 10.1371/journal.pone.0315280 (PMC11703119; doi:10.1371/journal.pone.0315280)
Supplement: S4 File — (ZIP) [file pone.0315280.s005.zip › S4.pdf]

Supporting information

S4. Real Data of Influenza. To validate the model, we have incorporated real influenza data from Mexico and Colombia. This data is provided in a separate Excel file named "Influenza Infected Data".
