# Supplementary material for: Bifurcation analysis of an influenza A (H1N1) model with treatment and vaccination
Source: PLoS One. 2025 Jan 6;20(1):e0315280. doi: 10.1371/journal.pone.0315280 (PMC11703119; doi:10.1371/journal.pone.0315280)
Supplement: Attachment — Submitted filename: Response_R1_PONE-D-24-32461.pdf [file pone.0315280.s006.pdf]

Md. Kamrujjaman, Ph.D.
Professor
Department of Mathematics
University of Dhaka
Dhaka-1000, Bangladesh
E-mail: kamrujjaman@gmail.com
kamrujjaman@du.ac.bd

September 29, 2024

Editor-in-Chief/Academic Editor
PLOS ONE

Dear Editor-in-Chief

Please find the revised version of the manuscript (Manuscript ID: PONE-D-24-32461) entitled: **"Bifurcation Analysis of an Influenza A (H1N1) Model with Treatment and Vaccination"** and consider it for publication in the journal **PLOS ONE**.

We have revised the manuscript according to the Editors/Reviewers comments and suggestions.

Authors' Note: The major corrections in the revised manuscript are highlighted in red for Reviewer 1 and in blue for Reviewer 2.

Reviewer-1

Reviewer Comments and Corresponding Answers:

1. In this article, the authors have studied a deterministic SVEIRT epidemic model for influenza, incorporating vaccination and treatment strategies. The model is proven to be well-posed, with the DiseaseFree Equilibrium (DFE) being globally asymptotically stable when the basic reproduction number, $\mathcal{R}_0$, is less than one, as demonstrated using Lyapunov functions and the LaSalle Invariance Principle. When $\mathcal{R}_0$ exceeds one, a unique Endemic Equilibrium (EE) exists, whose local and global stability are established through the Routh-Hurwitz criterion and non-linear Lyapunov functions, respectively. Moreover, numerical simulations of the model support the existence and stability of DFE and EE. The simulations indicate that higher transmission rates (β_1, β_2) and infection acquisition rates (α) lead to a rapid increase in disease burden. It is observed that if the rate of acquiring infection increases, then the disease burden will increase. Also, the growth rate in the $E(t)$ compartment raises the disease burden.

Meanwhile, treatment rate and vaccination rate can mitigate the factor $\mathcal{R}_0$ effectively. Furthermore, the contact rates β_1 and β_2 resulted in the rapid increase of the disease in the community. Also, the treatment of infected individuals in the acute phase is significant. Human recruitment rate can provide a great influence on controlling the disease.
This article may be publishable.
The following important references may be cited in the text:

- Stability analysis and optimal control of avian influenza virus A with time delays, International Journal of Dynamics and Control, 6(3), pp. 1351-1366 (2018). http://dx.doi.org/10.1007/s40435-017-0379-6
- Permanence and extinction for a nonautonomous avian-human influenza epidemic model with distributed time delay, Mathematical and Computer Modelling, 52(9-10), pp. 1794-1811 (2010). http://dx.doi.org/10.1016/j.mcm.2010.07.006
- Global Dynamics of a Nonautonomous SIRC Model for Influenza A with Distributed Time Delay, Differential Equations and Dynamical Systems, 18(4), pp. 341-362 (2010). http://dx.doi.org/10.1007/s12591-010-0066-y
- Dynamical behavior of SIRS model incorporating government action and public response in presence of deterministic and fluctuating environments. Chaos, Solitons and Fractals, 164 (2022) 112643. https://doi.org/10.1016/j.chaos.2022.112643
- Periodic transmission and vaccination effects in epidemic dynamics: a study using the SIVIS model. Nonlinear Dyn 112, 2381–2409 (2024). https://doi.org/10.1007/s11071-023-09157-4
- Modelling disease transmission through asymptomatic carriers: a societal and environmental perspective. Int. J. Dynam. Control (2024). https://doi.org/10.1007/s40435-024-01387-7
- Assessing the influence of public behavior and governmental action on disease dynamics: a PRCC analysis and optimal control approach. Eur. Phys. J. Plus 139, 527 (2024). https://doi.org/10.1140/epjp/s13360-024-05327-4
- Analysis of a delayed epidemic model with pulse vaccination. Chaos, Solitons & Fractals 66 (2014) 74–85. http://dx.doi.org/10.1016/j.chaos.2014.05.008

Authors' response: We thank you for the careful reading of the paper and for your reader-friendly suggestion. We have revised the manuscript and included relevant references based on your suggestions in reference lists 19-22. We have improved the literature review section by focusing on the main outcomes of the relevant studies mentioned above. Please check **page 4**. These changes are highlighted in red color in

the revised manuscript.
Please take a glance-
⋯ In a recent study, stability analysis and optimal control of avian influenza virus A with time delays is analyzed in [19]. Samanta et al. carried out mathematical and computer modeling along with permanence and extinction for a nonautonomous avian-human influenza epidemic model with distributed time delay [20]. Furthermore, the global dynamics of a nonautonomous SIRC model for influenza A with distributed time delay based on differential equations and dynamical Systems is studied effectively [21]. Meanwhile, in [22] Saha et al. have investi- gated the dynamical behavior of the SIRS model incorporating government action and public response in the presence of deterministic and fluctuating environments.

Reviewer-2

Reviewer Comments and Corresponding Answers:
After a thorough review, I believe the paper can be reconsidered after addressing the following points comprehensively:

1. Rewrite the first three lines of the abstract.
   Authors' response: Thank you for the suggestion. We have rewritten the first three lines of the abstract as-
   This research uses numerical simulations and mathematical theories to simulate and analyze the spread of the influenza virus. The existence, uniqueness, positivity, and boundedness of the solution are established. We investigate the fundamental reproduction number guaranteeing the asymptotic stability of equilibrium points that are endemic and disease-free. We also examine the qualitative behavior of the models ⋯

   All these revisions are highlighted in blue color in the revised manuscript.
2. There are several formatting issues. Once the terms "disease-free equilibrium (DFE)" and "endemic equilibrium (EE)" are introduced, consistently use the abbreviations throughout the text.
   Authors' response: The manuscript is revised accordingly and uses the abbreviations. All the revisions are highlighted in blue color. For example, please see **page 10, section 4** in the revised manuscript.
3. In the *Model Formulation* section, move the journal discussion to the *Introduction* and focus solely on the formulation aspects. Provide a detailed explanation of the transmission parameters and state variables.
   Authors' response: Accordingly, we have moved the journal discussion from *Model Formulation* section to *Introduction* section. In the revised manuscript this is highlighted in paragraphs 3, and 4 in blue colors.
   We admire your guidelines. The detailed explanation of the transmission parameters and state variables is now clearly stated in the model formulation section (**page 6**).

At a glance-

$\cdots$ In the SVEIRT model, $S(t)$ represents the susceptible population, which is subject to recruitment at rate Λ and can contract the disease through contact with exposed $E(t)$ and infectious $I(t)$ individuals at rates β_1 and β_2, respectively. Susceptibles may also be vaccinated at rate ϕ, or die naturally at rate μ. The vaccinated population $V(t)$ increases as individuals are vaccinated from $S(t)$, but they may still become exposed or infected at reduced rates due to vaccine efficacy ε, while also dying at rate μ. Exposed individuals $E(t)$ become infected at rate α or die at rate μ, having been infected by interaction with S or V. Infectious individuals $I(t)$ arise from the exposed class at rate α or through reduced infection from the vaccinated group, and they may either recover at rate γ, receive treatment at rate γ_1, or die due to natural and disease-related causes at rates μ and δ. Recovered individuals $R(t)$ gain immunity, leaving the infectious class at rate γ, while the treated population $T(t)$ increases due to treatment of infectious individuals at rate γ_1, with both classes experiencing natural mortality at rate μ.

4. Clearly state the assumptions imposed in the model formulation in this section.
   Authors' response: The assumptions imposed in the model are now clearly stated in **Section 2** of the revised manuscript. This is highlighted in blue color in the revised manuscript. At a glance-
   $\cdots$The SVEIRT model is based on several key assumptions. First, the total population is dynamic, with individuals entering the susceptible class through recruitment at a constant rate Λ and exiting all compartments due to natural death at rate μ. The disease is transmitted through contact between susceptible individuals and either exposed $E(t)$ or infectious $I(t)$ individuals, with transmission rates β_1 and β_2, respectively. Vaccination occurs at a constant rate ϕ, and the vaccine provides partial protection, reducing the susceptibility of vaccinated individuals by a factor of $1 - \varepsilon$. Exposed individuals progress to the infectious class at rate α, and infected individuals either recover at rate γ, receive treatment at rate γ_1, or die due to disease at rate δ. Recovered individuals are assumed to gain permanent immunity, while treated individuals are considered part of the infectious population but receive medical intervention. Finally, all compartments are subject to the same natural death rate μ, and demographic changes are not influenced by disease-related factors other than mortality in the infectious class.

5. Figure 2 is incomplete and lacks explanation. Include a detailed flow chart.
   Authors' response: The detailed flow chart of the model is now added in Figure 2 by highlighting the flow in each compartment (**page 8**). This is highlighted in blue color in the revised manuscript.

6. Discuss the biological feasibility and reference studies where, in the case of Influenza, a vaccinated person can still acquire the infection.
   Authors' response: We appreciate the reviewer's insightful suggestions. The manuscript has been revised accordingly and is highlighted in blue. For instance, please see **page 6, paragraph 2**, where the biological feasibility is added as follows:

Even though a person is vaccinated against Influenza, they can still acquire the infection due to several factors. The flu vaccine targets specific strains of the virus, but the virus mutates rapidly, leading to variations not covered by the vaccine. This phenomenon, known as "antigenic drift", can result in partial immunity or reduced vaccine effectiveness. Additionally, it takes about two weeks after vaccination for immunity to develop, leaving a window for infection. Lastly, individual immune responses to vaccines can vary, and some people may not build strong enough protection despite being vaccinated [1, 8, 10].

7. What about the waning of vaccine immunity in the model? Consider addressing this aspect.
   Authors' response: We have considered addressing this aspect in the revised manuscript and highlighted it in blue color in **page 6, last paragraph**. For instance:
   The waning of vaccine immunity can be reflected in the terms involving β_1 and β_2, which represent the transmission rates from exposed (E) and infectious (I) individuals. Specifically, the factor $(1 - \varepsilon)$ already accounts for the reduced susceptibility of vaccinated individuals. To model waning immunity, ε could be time-dependent, decreasing over time as immunity wanes. This would result in a gradual increase in susceptibility, thereby increasing the effective transmission rates β_1 and β_2 for the vaccinated population as immunity weakens.

8. Specify the values of β_1 and β_2 in Table 1, used for simulations.
   Authors' response: Thank you for providing thoughtful and reader-friendly guidance. We have used $\beta_1 = 0.0047$ and $\beta_2 = 0.0052$ for the simulations in Figure 6 and the rest of all figures, that were estimated. This is highlighted in blue color in the revised manuscript. Please check **page 41**.

9. All parameters used in the simulation are sourced from the literature, as indicated in Table 1. Clarify the purpose of using real data and parameter fitting.
   Authors' response: The purpose of using real data and parameter fitting in estimating the SVEIRT influenza model is to align the model's predictions with observed epidemiological trends. By fitting parameters such as transmission rates, recovery rates, and immunity loss rates to real-world data, the model can more accurately simulate the spread and control of influenza. This helps in understanding the dynamics of the disease and in making informed public health decisions for intervention strategies like vaccination and treatment.

10. Conduct a simulation analysis of the model based on estimated parameters.
    Authors' response: Thank you to the reviewer for the effective advice. The simulation analysis for the model is conducted in Figure 6 (**page 41**), for $\mathcal{R}_0 > 1$ and $\mathcal{R}_0 < 1$. We have also carried out the phase plane analysis of the model in Figures 7, 8, 9, and 10 based on estimated parameters in Table 1. All these are highlighted in blue color in the revised manuscript. Detailed discussions are added for each simulation accordingly. For instance, please check **pages 43, 44, 46, and 47** of the revised manuscript.

11. Suggest effective control parameters.

Authors' response: We appreciate this valuable advice. The effective control parameters are suggested based on phase plane analysis and highlighted in blue color in **page 47-48**. In a quick view:
$\cdots$ Based on phase plane analysis of the model, effective control can be achieved by manipulating key parameters. Increasing the vaccination rate (ϕ) shifts individuals from the susceptible (S) to the vaccinated (V) class, reducing the number of potential infections. Enhancing vaccine efficacy (ε) ensures that vaccinated individuals are less likely to become infected, limiting the spread. Reducing transmission rates (β_1, β_2) through interventions like social distancing and hygiene decreases the rate at which susceptible and vaccinated individuals come into contact with exposed or infected individuals. Additionally, improving the treatment rate (γ_1) shortens the infectious period, lowering the disease burden in the population. Together, these adjustments stabilize the system, minimizing infection peaks and leading to more effective disease control.

12. Estimate parameters and R_0 based on real data.
Authors' response: We have estimated the parameters based on the case study in Mexico and Colombia data in sections 9 and 10. All the estimated parameters are summarized in Table 2, **page 52** in the revised manuscript. Meanwhile, we have estimated the threshold quantity $\mathcal{R}_0$ in contour plot and box plot analysis in sections 8.4 and 8.5, respectively. Please see **pages 48 and 51** where the simulations are highlighted in blue. A detailed discussion of the numerical analysis is also added. These results reflect that, by varying the effective control parameters β_1, β_2, α, γ_1, and γ, the basic reproduction number lies in a range $[1.5, 4.5]$.

13. In Theorem 5, how is $dU_1/dt < 0$? This result is ambiguous. Please review it carefully.
Authors' response: Thank you for the nice observation. In Lyapunov function stability analysis, the condition $\frac{dU_1}{dt} < 0$ indicates that the Lyapunov function U_1 is decreasing over time, suggesting convergence towards equilibrium. This negative derivative often arises from dissipative terms, such as damping or recovery mechanisms, which help reduce deviations from equilibrium. Thus, $\frac{dU_1}{dt} < 0$ confirms that the equilibrium is globally asymptotically stable. This illustrates the efficacy of the Lyapunov method in assessing long-term system stability.
The proof of the Theorem 5 is revised and attached in Appendix A. For instance, please check **page 63** of the revised manuscript. All the conditions are highlighted in blue color in the revised manuscript.

14. Analyze the impact of the vaccination parameter on the reproduction number and, ultimately, on disease incidence.
Authors' response: This analysis is performed in Figures 6, 7, 8, 9, and 10 in the revised manuscript and highlighted in blue color. The key points are summarised in **pages 47, 48** in the revised manuscript and highlighted in blue color.

15. From Table 2, $\beta_1 > \beta_2$, meaning the transmission probability from the exposed class is greater than the infected class. Explain the rationale behind this.
Authors' response: There was a typographical error, which has now been carefully revised. All simulations have been conducted using the estimated parameters from

Table 2. The condition will be $\beta_2 > \beta_1$, and the careful correction has been highlighted in blue color in the revised manuscript, **page 52** in Table 2. This reflects that individuals in the infected class are more contagious than those in the exposed class. Infected individuals typically have a higher viral load and are often symptomatic, making them more likely to transmit the disease. In contrast, exposed individuals are in the incubation phase with a lower viral load and are less likely to spread the infection. This difference aligns with the epidemiological understanding that symptomatic individuals are usually more responsible for transmission than those who are asymptomatic or pre-symptomatic.

General: We thank all reviewers for their constructive suggestions and comments to improve the quality of the paper in the Acknowledgment section.

Yours sincerely,

Md. Kamrujjaman

Editor-in-Chief/Academic Editor
PLOS ONE
